# PI4-kinase A is essential for survival of the GnRH neurons

**DOI:** 10.3389/fendo.2026.1813498

**Published:** 2026-05-05

**Authors:** Stephanie Constantin, Naseratun Nessa, Stanko S. Stojilkovic

**Affiliations:** Section on Cellular Signaling, The Eunice Kennedy Shriver National Institute of Child Health and Human Development, National Institutes of Health, Bethesda, MD, United States

**Keywords:** GnRH neurons, kisspeptin neurons, development, neuronal survival, phosphatidylinositol 4-kinase alpha, PI4P, PI(4,5)P2, PI(3, 4, 5)P3

## Abstract

The signaling pathways that control embryonic development and migration of gonadotropin-releasing hormone (GnRH) neurons, as well as the postnatal fate and function of differentiated cells, are the subject of ongoing research. Here, we examined the role of phosphoinositides in this complex multistep process by generating GnRH neuron-specific phosphatidylinositol 4-kinase alpha knockout mice. The knockout mice were indistinguishable from their control littermates at all time points studied. However, adult knockout females and males were infertile, as reflected by the absence of GnRH immunoreactivity and *Gnrh1* expression, and reduced expression of pituitary gonadotroph-specific genes, accompanied by underdeveloped gonads and reproductive organs. Kisspeptin immunoreactivity was preserved and *Kiss1* expression was modified in a nucleus-specific-manner, consistent with the loss of circulating sex steroid hormones. Embryonic neurogenesis and migration of GnRH neurons were not dramatically impaired, as evidenced by normal *Gnrh1* expression in the hypothalamus of neonatal animals and the presence of immunoreactive GnRH neurons in infantile mice in comparable distribution to age-matched controls. However, their cellular degeneration was observed in infantile mice, accompanied by reduced *Gnrh1* expression. GnRH neuron-specific expression of tdTomato confirmed their postnatal degeneration, leading to their death, while ectopic tdTomato-positive cells located in the lateral septum remained unaffected. These findings indicate that phosphatidylinositol 4-kinase alpha activity is not critical for the establishment of the GnRH neuronal system, in contrast to the survival of GnRH neurons.

## Significance statement

Differentiation of neuroendocrine GnRH cells involves neurogenesis in the olfactory placodes, migration to the hypothalamus, projection to the median eminence, and connections with upstream neurons, including kisspeptin neurons. Here we show that knockout of phosphatidylinositol 4-kinase alpha in GnRH neurons does not prevent the establishment of the GnRH neuronal system. However, the activity of this enzyme is essential for survival of differentiated GnRH neurons; in the absence of this gene, the neurons die, causing infertility in both female and male mice.

## Introduction

Gonadotropin-releasing hormone (GnRH)-secreting neurons originate in the medial olfactory placodes and migrate to the preoptic area (POA)/hypothalamus during embryonic development (1). Their cell bodies form a rostrocaudal continuum from the preoptic area to the mediobasal hypothalamus, with the highest cell density around the organum vasculosum lamina terminalis (OVLT) and projecting to the median eminence. During *in utero* development, connections between GnRH neurons and kisspeptin neurons are established in the arcuate nucleus (ARN) (2). Before the onset of puberty, connections between these two cell types are also established in the rostral periventricular area of the third ventricle (RP3V) (3), as the responsiveness of GnRH neurons to kisspeptin increases (4). Kisspeptin neurons in the ARN, also known as KNDy neurons, co-express neurokinin B and dynorphin A (5). These neurons act as GnRH pulse generator by innervating distal projections of GnRH neurons in the median eminence in both females and males (6), and their function is inhibited by gonadal steroids. RP3V kisspeptin neurons project to GnRH cell bodies in the preoptic area and to the distal projections of GnRH neurons (6) and act as GnRH surge generator. Unlike KNDy neurons, the function of the RP3V kisspeptin neurons is stimulated by gonadal steroids, leading to preovulatory GnRH release (7). Pulsatile release of GnRH stimulates expression of the pituitary gonadotropin genes *Fshb* and *Lhb*, their protein synthesis, and the release of follicle-stimulating hormone and luteinizing hormone. These hormones then control gametogenesis and steroidogenesis in the gonads of both sexes and ovulation in females (8).

The signaling pathway involved in communication between kisspeptin neurons and GnRH neurons is well characterized. Kisspeptin receptors expressed in GnRH neurons signal via the Gq/phospholipase C pathway, which leads to the hydrolysis of phosphatidylinositol 4,5-bisphosphate (PI(4,5)P2) to inositol 1,4,5-trisphosphate and diacylglycerol (9). This is followed by the initiation of inositol 1,4,5-trisphosphate receptor-dependent release of calcium from intracellular pools and modulation of potassium and nonselective cation channels (10). This causes depolarization of GnRH neurons accompanied by an increase in firing frequency (11) and/or facilitation of voltage-gated calcium influx (12). A decrease in PI(4,5)P2 levels during this signaling cascade prevents the subsequent kisspeptin response, and this refractory period can be reduced by replenishing PI(4,5)P2 (13). Therefore, PI(4,5)P2 is a key player and regulator of the kisspeptin-induced response in GnRH neurons. However, the roles of this and other phosphoinositides in the development of the GnRH neuronal system and its function have not been studied.

Phosphoinositides are formed by the regulated activity of phosphoinositide kinases and phosphatases. Among these enzymes, phosphatidylinositol 4-kinase alpha (PI4KA) is responsible for the formation of phosphatidylinositol 4-phosphate (PI4P) in the plasma membrane, which is itself a signaling molecule and the starting point for the formation of PI(4,5)P2. This phosphoinositide is vital for numerous cellular functions, including phospholipase C signaling, ion channel gating, and control of exocytotic pathways. Furthermore, phosphorylation of PI(4,5)P2 by class I phosphatidylinositol-3-kinase generates phosphatidylinositol 3,4,5-trisphosphate (PI(3,4,5)P3), which exerts its intracellular messenger functions by binding to numerous effectors containing a pleckstrin homology domain (14). PI4KA is also well expressed in GnRH neurons (15), but its specific function has not been studied in these cells. Global knockout of PI4KA in mouse models is embryonically lethal (16), but cell type–specific PI4KA knockout experiments offer the potential to study the effects of this gene on specific cells function (17). Here, we present data obtained from experiments with GnRH neuron-specific knockout mice to determine the potential role of PI4KA in these cells.

## Material and methods

### Animals

All experimental procedures were approved by the National Institute of Child Health and Human Development, Animal Care and Use Committee (Protocol 22-041). Pi4ka-LoxP mice (MGI:6294260) (18) were crossed with Rosa-tdTomato mice (MGI:3813512) (19) to generate dams Pi4ka^LoxP/LoxP^ Rosa^tdTom/tdTom^. Pi4ka-LoxP mice were crossed with Gnrh1-cre mice (MGI: 3691288) (20) to generate Gnrh1^Cre/Cre^ Pi4ka^LoxP/wt^ bucks. The breeding of these dams and bucks resulted in experimental animals i.e. conditional knockout mice in which PI4KA was specifically inactivated in GnRH neurons (Gnrh1^Cre/wt^ Pi4ka^LoxP/LoxP^ Rosa^tdTom/wt^; hereafter knockouts) and their control littermates (Gnrh1^Cre/wt^ Pi4ka^LoxP/wt^ Rosa^tdTom/wt^; hereafter controls). Cre-dependent recombination could be monitored with tdTomato expression in both knockouts and controls. Fertility was assessed as follows: three to seven knockout and control mice (7–12-week-old) were mated with wild-type C57/Bl6 of the opposite sex for <4 weeks to assess their fertility. The time of the first litter, relative to the date of the mating, was recorded. Necropsy was performed at postnatal day (PND) 45 on control and knockout mice.

### qRT-PCR analysis

Neonatal (PND3), infantile (PND 9 and PND10), juvenile (PND15 and PND20), peripubertal (PND45), and adult (8 to 25 weeks) females and males were used to collect hypothalamic and pituitary tissues. Before PND10, the whole hypothalamus was collected. From PND10, the hypothalamus was divided into rostral and caudal parts using the optic tract as a demarcation. Whole pituitaries were collected simultaneously. All tissues were snap frozen on dry ice. Hypothalamic and pituitary RNA were isolated using the RNeasy Plus Mini Kit (Qiagen, Valencia, CA), and reverse transcription was performed using the Transcriptor First Strand cDNA Synthesis Kit (Roche Applied Sciences, Indianapolis, IN), according to the manufacturer’s respective protocols. Applied Biosystems predesigned TaqMan Gene Expression Assays (Applied Biosystems, Waltham, MA) were used for the following genes: *Gapdh* (Mm99999915_g1), *Gh1* (Mm00433590_g1), *Gnrh1* (Mm01315604_m1), *Gnrhr* (Mm00439143_m1), *Kiss1* (Mm03058560_m1), *Lhb* (Mm01205505_g1), *Mkrn3* (Mm00844003_s1), *Pdyn* (Mm00457573_m1), *Pomc* (Mm00435874_m1), *Prl* (Mm00599950_m1), *Spp1* (Mm00436767_m1), *Tac2* (Mm01160362_m1), and *Tshb* (Mm03990915_g1).

### Immunolabeling

Mice, anesthetized with a ketamine/xylazine cocktail (200/20 mg/kg), were transcardially perfused with 0.1 M phosphate buffer saline (PBS), followed by PBS containing 4% formaldehyde. Brains were postfixed overnight and then transferred to 30% PBS containing sucrose for 2 nights. Brains were snap-frozen on dry ice using Tissue-Plus OCT (Fisher Healthcare/ThermoFisher Scientific) and stored at −80 °C until sectioning. Adult brains were cut into four sets of 40 µm coronal sections using a Leica SM2010 R sliding microtome (Wetzlar, Germany). PND10 brains were cut into two sets of 50 µm coronal sections. All sections were stored at −20 °C in cryoprotectant until staining (>5 days). For immunochemistry, PND10 and adult free-floating sections were processed for GnRH and kisspeptin with rabbit anti-GnRH antibody (RRID: AB_572248; dilution 1:15,000) or kisspeptin antibody (RRID: AB_2314709; dilution 1:10,000), as previously described (21). For immunofluorescence, PND10 and adult free-floating sections were incubated for 1 h at room temperature in blocking solution (10% normal goat serum plus 0.3% Triton X-100), washed several times in PBS, and incubated (2–3 nights, 4 °C) in guinea pig anti-GnRH antibody (RRID: AB_2721118). Sections were then washed in PBS, incubated (1 h, room temperature) with a secondary AlexaFluor 488 goat anti-guinea pig antibody (AB_2534117; 1:1,000 in PBS/0.3% Triton X-100). All images were captured using 10X and 40X magnification objectives on an Eclipse Ti2 microscope (Nikon, Melville, NY). Excitation was provided by a SOLA V-nIR light system (Lumencor, Beaverton, OR), and emission was captured by an ORCA-Flash4.0 V3 digital camera (Hamamatsu, Bridgewater, NJ). Images were processed using FIJI (22) and montages were created in Photoshop (Adobe, San Jose, CA).

### Statistical analysis

Results are presented as representative histological images or mean ± SEM values from at least three similar experiments, and the number of replicates (N) is indicated in Results, Figures, and/or Figure Legends. Graphs were generated and statistically analyzed using the KaleidaGraph program (Synergy Software, Reading, PA) or GraphPad Prism (San Diego, California) with one-way ANOVA and *post hoc* multiple comparison test for data containing more than two groups, with at least P < 0.01 considered statistically significant. Figures were finalized using Adobe Photoshop and Illustrator (Adobe, San Jose, CA).

## Results

### GnRH neuron-specific PI4KA knockout mice are infertile

The experiments were conducted with female and male wild-type, control, and knockout mice. Physically, knockout mice were indistinguishable from their control littermates. After housing with wild-type mice of the opposite sex, control females had their first litter within 26 ± 3 days (N = 5 breeding pairs) and control males had their first litter within 23 ± 2 days (N = 7 breeding pairs). However, neither knockout females nor males (N = 3 breeding pairs each sex) had litters when mated with wild-type mice of the opposite sex for >4 weeks.

**Figure 1:**
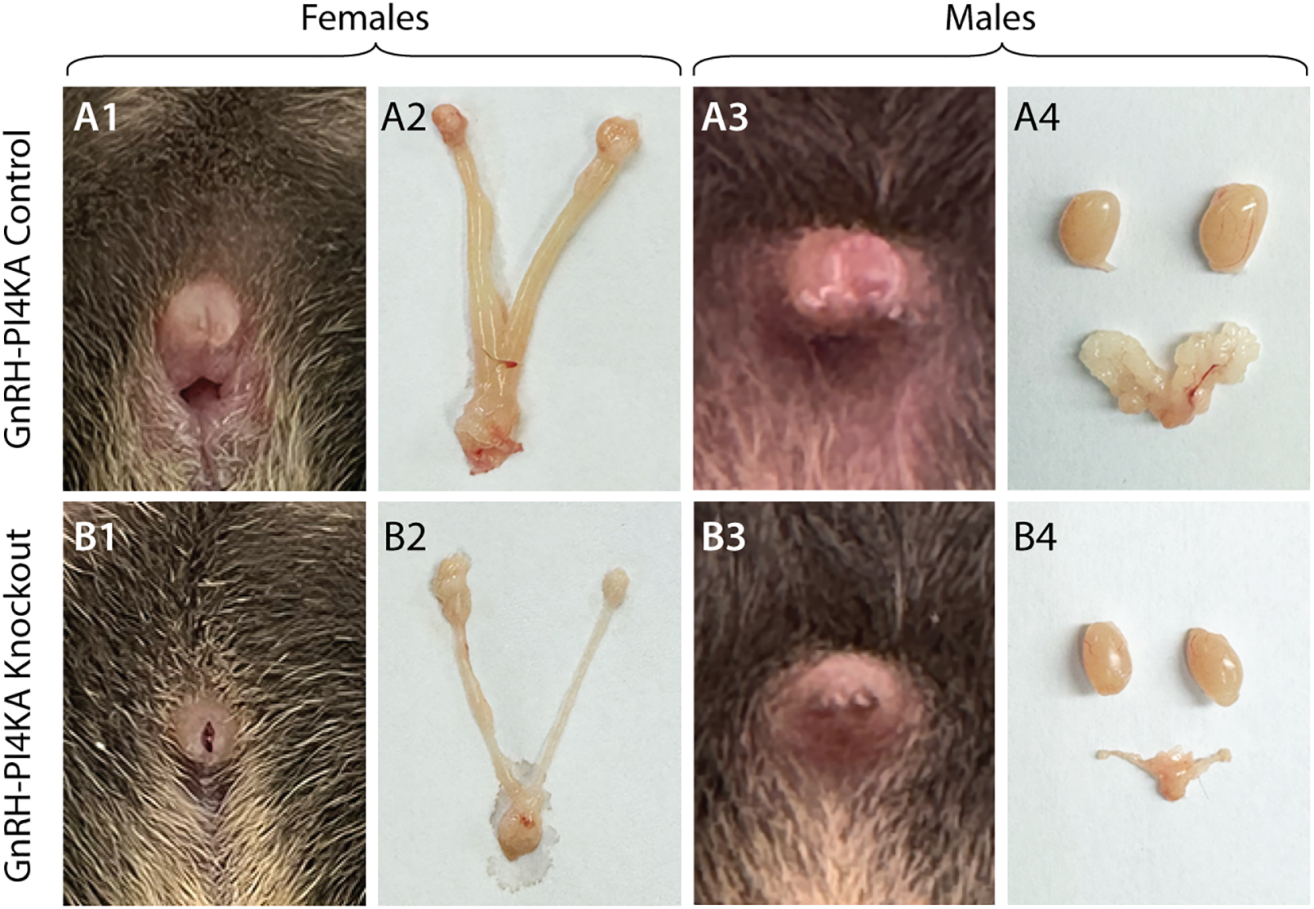
Comparison of reproductive organs of control and knockout mice at seven weeks of age. In control females, the vagina was perforated **(A1)** and the animals had a normally developed uterus and ovaries **(A2)**. Control males showed foreskin separation **(A3)**, and the testes and seminal vesicles were normally developed **(A4)**. In contrast, female knockouts showed imperforate vaginas **(B1)** and underdeveloped uterus and ovaries **(B2)**. Male knockouts did not show foreskin separation **(B3)**, and the testes and seminal vesicles were atrophied **(B4)**.

The infertility of the knockouts was associated with the status of their reproductive organs, as shown in **Figures 1A1, A2, B1, B2** for females and **Figure 1A3, A4, B3, B4** for males; controls (top panels) and knockouts (bottom panels), all PND45. In contrast to controls, knockouts lacked external features typically associated with sexual maturity; knockout females did not show a vaginal opening (**Figures 1A1 vs B1**) and males did not show separation of the prepuce, also known as foreskin separation (**Figures 1A3 vs B3**). Necropsy further revealed that knockout females (**Figures 1A2 vs B2**) and males (**Figures 1A4 vs B4**) displayed underdeveloped reproductive organs compared with their control littermates. These data clearly indicate that the hypothalamic-pituitary-gonadal axis is not operational in PI4KA knockouts.

### Loss of GnRH expression causes infertility of knockout mice

**Figure 2:**
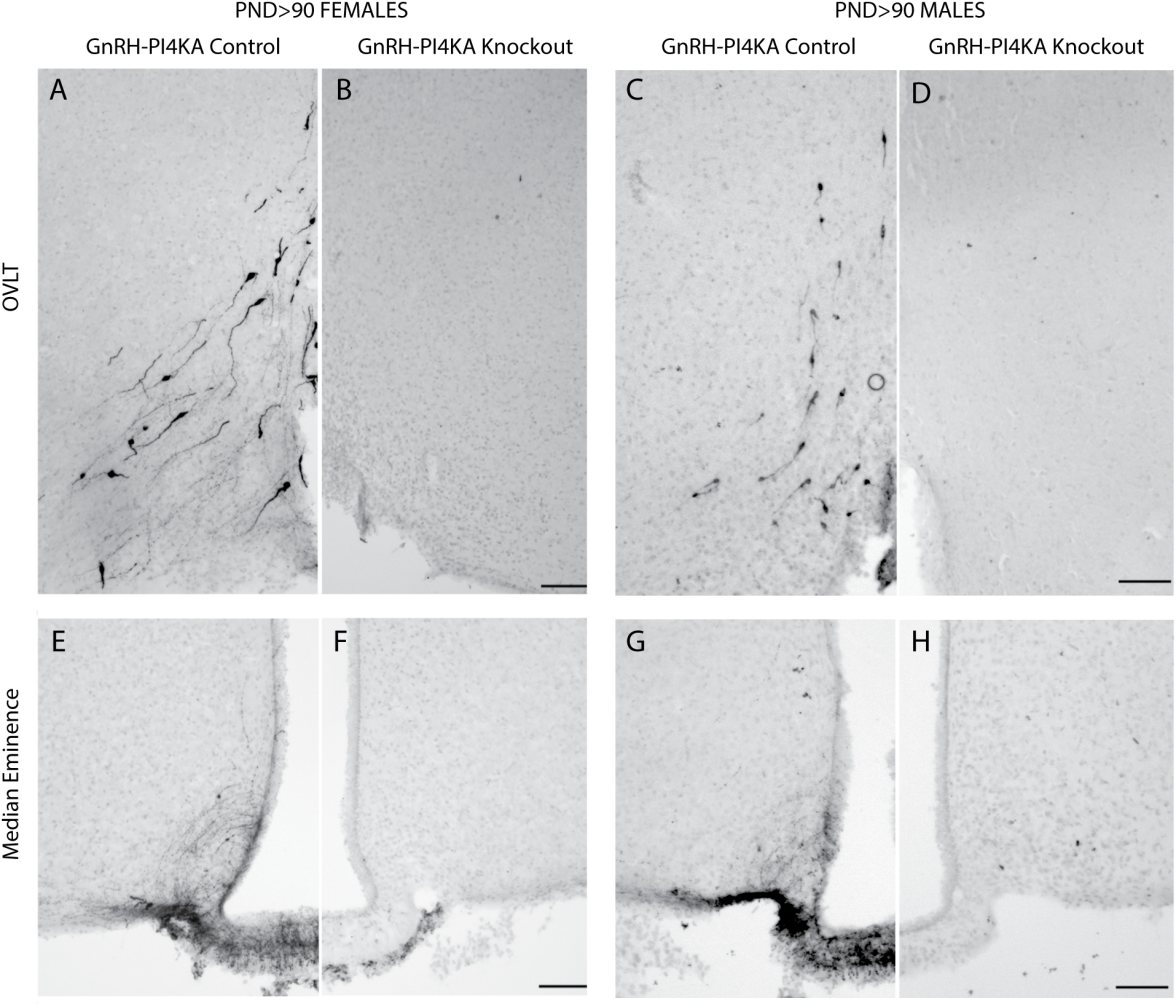
Immunostaining of GnRH-expressing neurons in hypothalamic tissue of adult control and knockout mice. **(A–D)**, GnRH-expression pattern in the organum vasculosum lamina terminalis (OVLT) of control **(A)** and knockout **(B)** females, and control **(C)** and knockout **(D)** males. Typical bipolar GnRH-positive neurons were present in control females and males, but were not visible in the OVLT area of knockouts. **(E–H)**, GnRH expression pattern in the medial eminence of control **(E)** and knockout **(F)** females, and control **(G)** and knockout **(H)** males. Typical GnRH-positive fibers were detected in the medial eminence of controls, but not of knockouts. Horizontal bars at 100 µM. Two mice were examined for each genotype/sex.

Because PI4KA knockout is specific for GnRH-secreting neurons, which are essential for the establishment and function of the hypothalamic-pituitary-gonadal axis, in further experiments we performed immunochemical staining to qualitatively assess GnRH peptide expression in hypothalamic tissue of adult control and knockout mice as described in Material and Methods. Immunostaining was examined across the rostro-caudal GnRH continuum. For representation, we focused on GnRH immunoreactivity around the OVLT area (**Figures 2A–D**), which contains many GnRH neurons. Adult control females (**Figure 2A**) and males (**Figure 2C**) showed a typical distribution of GnRH-expressing cell bodies (N = 2, each sex). In contrast, knockout females (**Figure 2B**) and males (**Figure 2D**) had no GnRH immunoreactivity (N = 2, each sex).

We also analyzed GnRH immunoreactivity in median eminence of adult control and knockout animals (**Figures 2E–H**). In general, GnRH neurons project long processes to the median eminence at the base of the hypothalamus, where they branch extensively and form terminals near blood vessels to release the hormone into the pituitary portal system (23). GnRH immunoreactivity highlighted fibers in control females and males (**Figures 2E, G**), but not in knockout females and males (**Figures 2F, H**).

**Figure 3:**
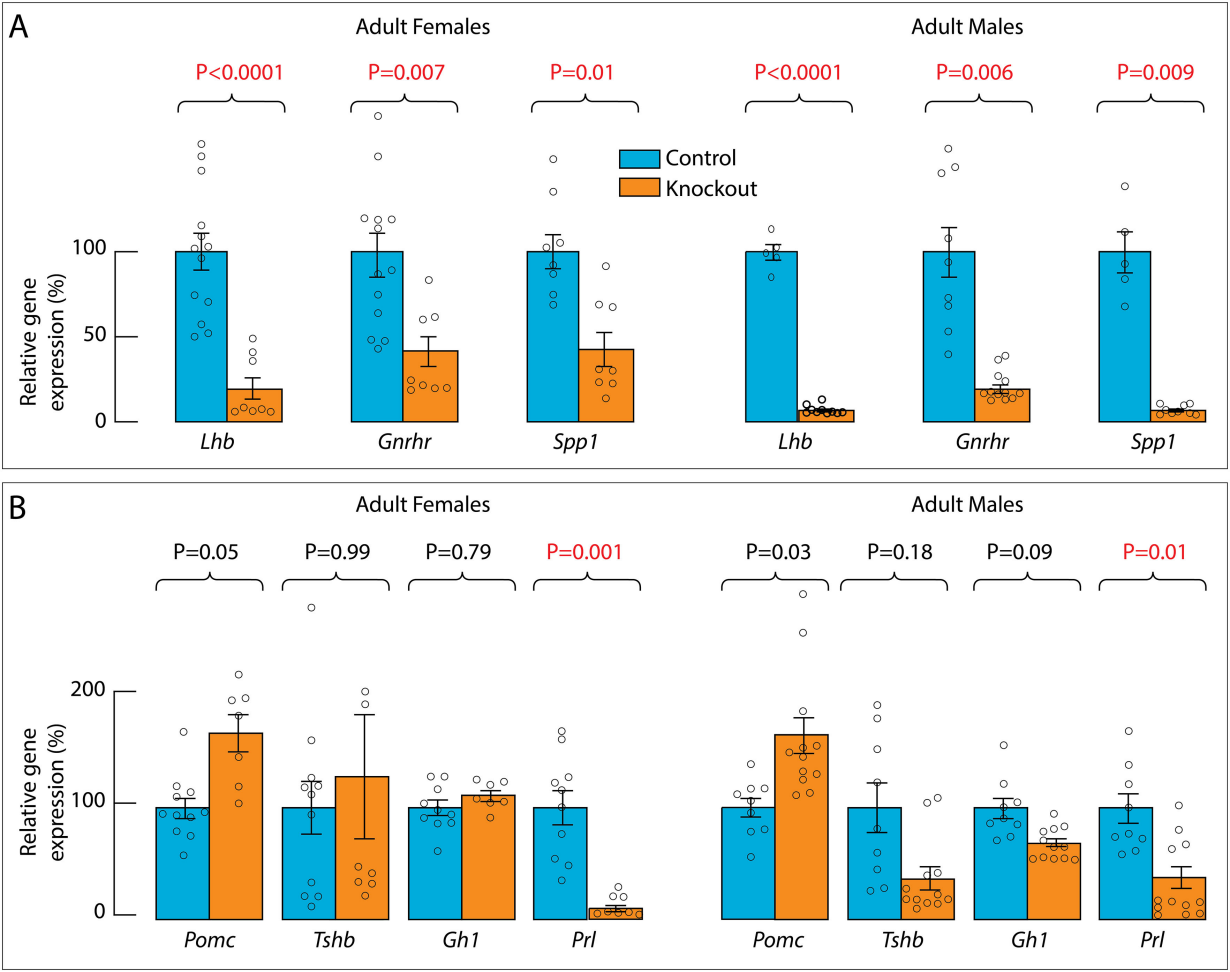
Expression of pituitary genes in adult control and knockout mice. **(A)**, Expression of gonadotroph-specific genes *Lhb*, *Gnrhr*, and *Spp1*. **(B)**, Expression of corticotroph/melanotroph-specific *Pomc*, thyrotroph-specific *Tshb*, somatotroph-specific *Gh1*, and lactotroph-specific *Prl.* Gene expression was assessed by qRT-PCR using whole pituitary for mRNA extraction, values for controls are shown as 100%, and for knockouts as percentage change relative to controls. Data are presented as means ± SEM, statistical analysis was performed using data before normalization, and P values were calculated using Welch’s ANOVA followed by *post hoc* Dunnett’s T3 multiple comparisons. Individual data points for each mouse included in this analysis are represented by small circles within and above the bars. Red numbers indicate significant differences between pairs.

By binding to its receptor in pituitary gonadotrophs, GnRH controls the expression of gonadotroph-specific *Lhb*, *Fshb*, and *Gnrhr* (8). Therefore, the expression of these genes in the pituitary can be used as an indicator of the status of GnRH secretion. Consistent with the lack of immunoreactivity for GnRH, the expression of *Lhb* and *Gnrhr* was significantly reduced in knockout females and males compared with adult controls. The expression of *Spp1*, another gonadotroph-specific gene within the pituitary cell populations (24), was also significantly reduced in knockout females and males (**Figure 3A**). Expression of *Pomc*, *Tshb*, and *Gh1*, the marker genes of corticotroph/melanotrophs, thyrotrophs and somatotrophs, respectively, was unaffected, while expression of *Prl*, a marker gene of lactotrophs, was significantly reduced in knockout females and males (**Figure 3B**). Together, the lack of immunoreactive GnRH neurons and the reduced expression of *Lhb* and *Gnrhr* in knockouts provide a rationale for the underdeveloped reproductive organs and infertility of these animals, and the concomitant reduction in gonadal steroidogenesis leads to a decrease in *Spp1* and *Prl* expression.

### Kisspeptin immunoreactivity is preserved in adult knockout mice

**Figure 4:**
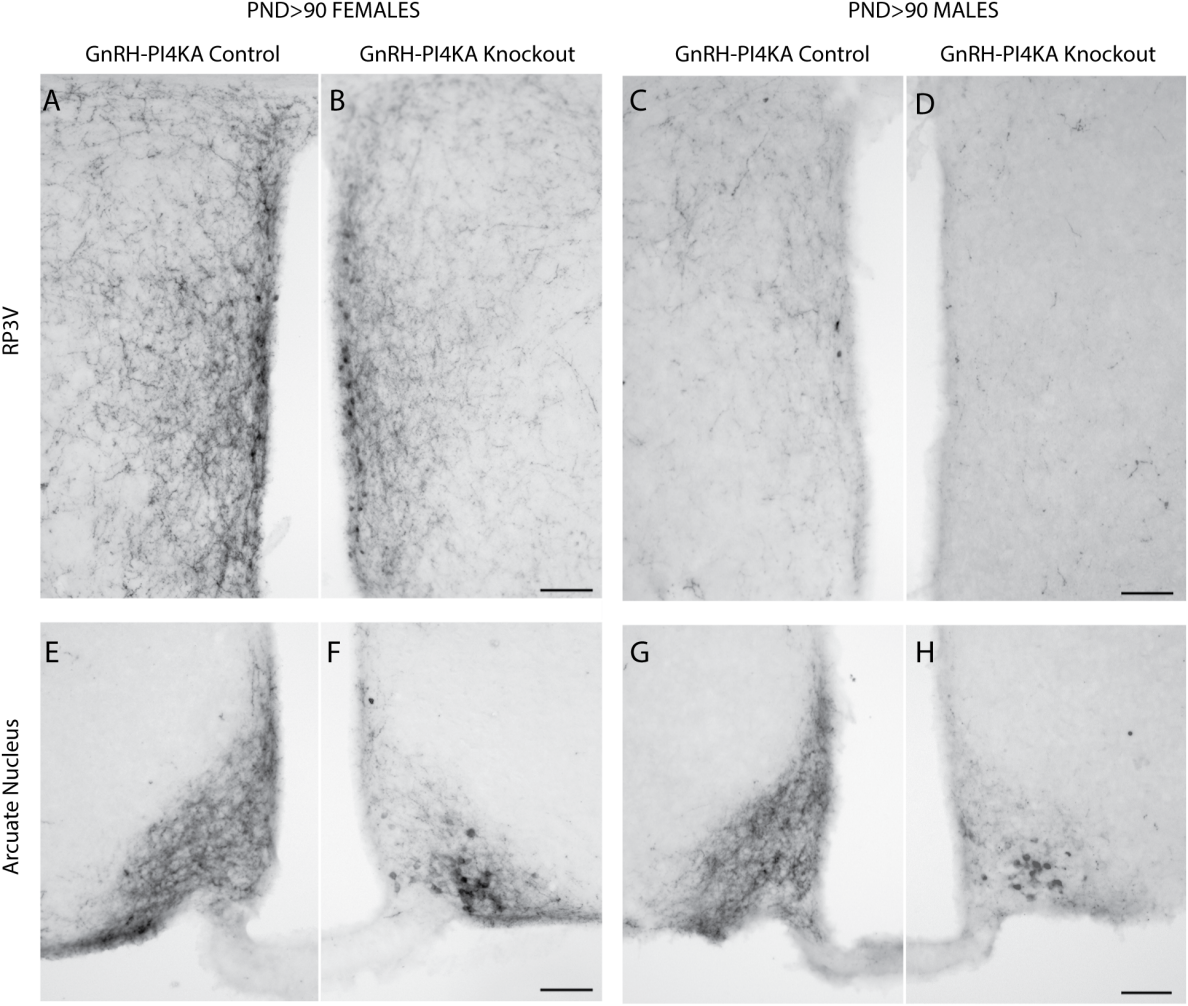
Kisspeptin immunoreactivity in hypothalamic tissue of adult control and knockout mice. **(A–D)**, The expression pattern of kisspeptin in the rostral periventricular area of the third ventricle (RP3V) of control **(A)** and knockout **(B)** females, and of control **(C)** and knockout **(D)** males. **(E–H)**, The expression pattern of kisspeptin in the arcuate nucleus of control **(E)** and knockout **(F)** females, and of control **(G)** and knockout **(H)** males. Horizontal bars at 100 µM. The same mice used for GnRH staining were also used for kisspeptin staining.

It is well known that kisspeptin neurons provide a key stimulatory input to GnRH neurons (25), and that *Kiss1* expression is stimulated or inhibited by circulating sex steroid hormones, depending on the specific brain region (26, 27). The lack of detectable GnRH immunoreactivity in infertile knockouts prompted us to analyze the status of kisspeptin neurons in the same animals used for GnRH immunostaining. **Figure 4** shows that both controls and knockouts express two kisspeptinergic subpopulations: one located in RP3V (A - D) and another in the ARN (E - H). RP3V kisspeptin expression was sexually dimorphic; it was highly expressed in control females (**Figures 4A, B**) and weakly expressed in males (**Figures 4C, D**). The density of fibers was reduced in knockout females, but the reduction was less pronounced in males due to minimal RP3V kisspeptin immunoreactivity. In contrast, ARN kisspeptin expression was not sexually dimorphic but was affected by PI4KA knockout in GnRH neurons of females (**Figures 4E vs F**) and males (**Figures 4G vs H**). In both sexes, controls showed fiber-rich immunoreactivity in ARN without identifiable kisspeptin cell bodies, whereas knockouts showed fiber-poor immunoreactivity with readily identifiable kisspeptin cell bodies.

### Loss of *Gnrh1* expression coincides with morphological changes in GnRH neurons

**Figure 5:**
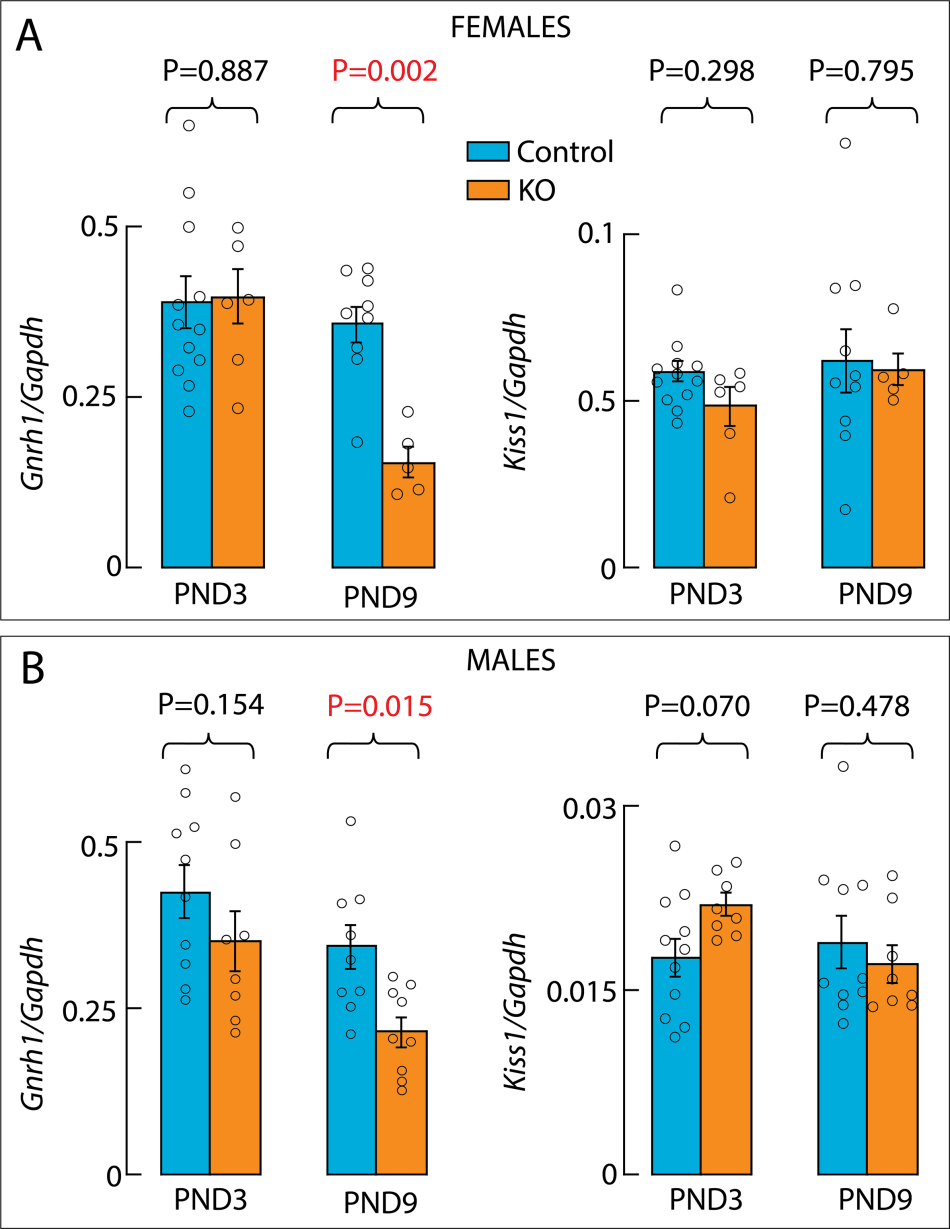
qRT-PCR analysis of Gnrh1 and Kiss1 gene expression in the hypothalamus of control (blue) and knockout (orange) mice. **(A, B)**, *Gnrh1* (*left*) and *Kiss1* (*right*) expression assessed in the whole hypothalamus of neonatal (PND3) and infantile (PND9) controls and knockout females **(A)** and males **(B)**. Data are presented as means ± SEM and numbers in parentheses indicate the number of animals per group. P values were calculated using one-way ANOVA followed by *post hoc* Fisher’s least significant difference as described in Methods. Red numbers indicate significant differences. Individual data points for each mouse included in this analysis are represented by small circles within and above the bars.

The differentiation of GnRH neurons in the olfactory placodes and their migration to the POA/hypothalamus are restricted to embryonic life (1). Therefore, the loss of GnRH peptide expression in adult knockout mice may reflect a loss of neurogenesis, or neuronal migration embryonically, or changes in neuronal survival. To address this issue, we initially studied *Gnrh1* expression by qRT-PCR using whole hypothalamic tissue from neonatal animals (PND3) and infantile animals (PND9). At PND3, *Gnrh1* expression was comparable in control (N = 12) and knockout (N = 6) females but was significantly reduced at PND9 in knockout (N = 5) females compared to control females (N = 9), while *Kiss1* expression remained unchanged at both ages (**Figure 5A**). The same conclusion was reached in experiments with PND3 (N = 10 controls; N = 8 KOs) and PND9 (N = 9 controls; N = 9 KOs) control and knockout males (**Figure 5B**).

**Figure 6:**
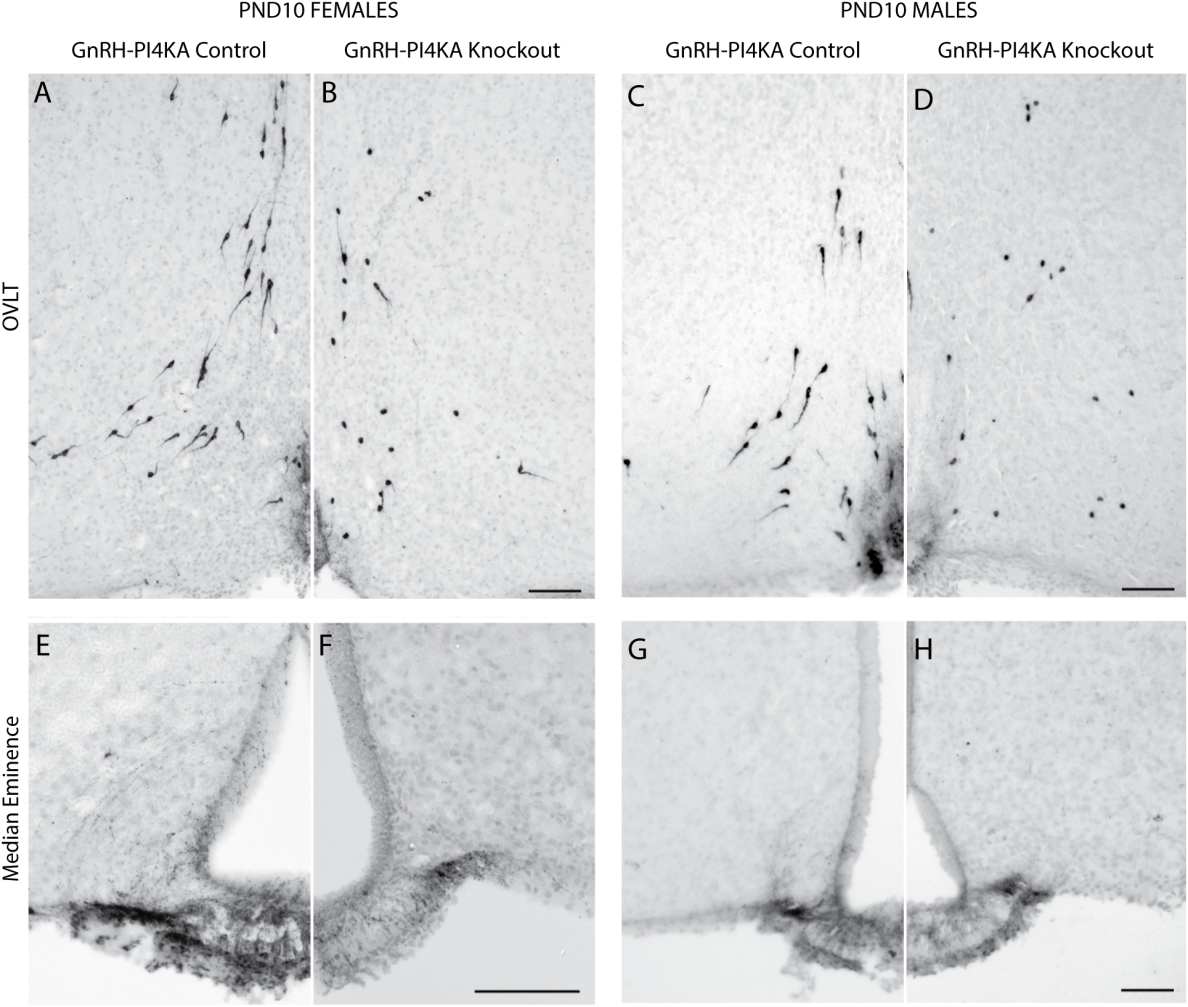
Immunostaining of GnRH neurons in hypothalamic tissue from infantile control and knockout mice. **(A–D)**, GnRH-positive neurons in the OVLT hypothalamic area of control **(A)** and knockout **(B)** females, and control **(C)** and knockout **(D)** males. Note the typical distribution of bipolar GnRH-positive neurons in control females and males. GnRH-positive neurons in knockouts showed a similar distribution pattern, but cells were predominantly round. **(E–H)**, GnRH expression pattern in the medial eminence of control **(E)** and knockout **(F)** females, and control **(G)** and knockout **(H)** males. It should be noted that GnRH immunoreactivity was reduced in medial eminence of knockouts. Horizontal bars at 100 µM. Two mice were examined for each genotype/sex.

In further experiments, GnRH immunoreactivity was qualitatively assessed at PND10 when Gnrh1 expression was significantly reduced but not abolished in knockouts compared to age-matched controls. As in adult controls (**Figure 2**), this analysis was illustrated with GnRH immunoreactivity around the OVLT (**Figures 6A–D**), and in the median eminence (**Figures 6E–H**). Control females (**Figure 6A**) and males (**Figure 6C**) (N = 2, each sex) showed a distribution of unipolar and bipolar neurons in the POA/hypothalamus comparable to adult controls (**Figures 2A, C**). PND10 knockouts (N = 2, each sex) displayed a similar distribution of GnRH-immunoreactive cells (**Figures 6B, D**), in contrast to adult knockouts (**Figures 2B, D**). However, in PND10 knockouts, most GnRH cell bodies appeared round without processes. Furthermore, GnRH immunoreactivity was present but appeared reduced in the median eminence of knockouts, compared with age-matched controls (**Figures 6E–H**).

While these observations do not rule out subtle changes in GnRH neurons during embryonic development, the successful migration of most PI4KA-deficient GnRH neurons to the POA/hypothalamic region, their ability to target the median eminence, and similar *Gnrh1* expression in control and knockout mice at PND3 indicate that the establishment of the GnRH neuronal system is not dramatically impaired in knockouts. However, reduced *Gnrh1* expression at PND9, as well as the morphological changes of GnRH neurons in knockout mice at PND10, suggest that major changes in PI4KA-deficient GnRH neurons occur postnatally. Since morphological changes of GnRH neurons were concomitant with the reduction in *Gnrh1* expression, qRT-PCR analysis could be used to quantitatively assess the timeline of postnatal changes in the GnRH neuronal system in a larger cohort of mice.

### Effects of knockout on *Gnrh1* and *Kiss1* expression during development

In further experiments, we measured levels of gene expression at different postnatal time points in control and knockout mice to assess changes in the GnRH neuronal system. The hypothalamic tissue was divided into rostral and caudal parts to examine the kinetics of postnatal *Gnrh1* and *Kiss1* expression in the two regions. Experiments were conducted with females and males aged PND 10, 15, 20, 45 and 90. To ensure that the landmarks we used for dissection allowed us to distinguish region-specific expression, we assessed *Gnrh1* expression in the rostral region (including the OVLT+RP3V areas) and the caudal region (including the ARN). As expected, Welch’s ANOVA, followed by *post hoc* Dunnett’s T3 multiple comparison test, confirmed that *Gnrh1* expression was significantly higher in the rostral hypothalamus than in the caudal hypothalamus in both females (1.09 ± 0.14 vs. 0.20 ± 0.06; P = 0.0005) and males (0.80 ± 0.07 vs. 0.15 ± 0.06; P = 0.003). Furthermore, *Kiss1* expression was significantly higher in the rostral hypothalamus in females than in males (0.36 ± 0.05 vs. 0.04 ± 0.004; P = 0.001), but was similar in the caudal hypothalamus in females and males (0.11 ± 0.03 vs. 0.05 ± 0.01; P = 0.22).

**Figure 7:**
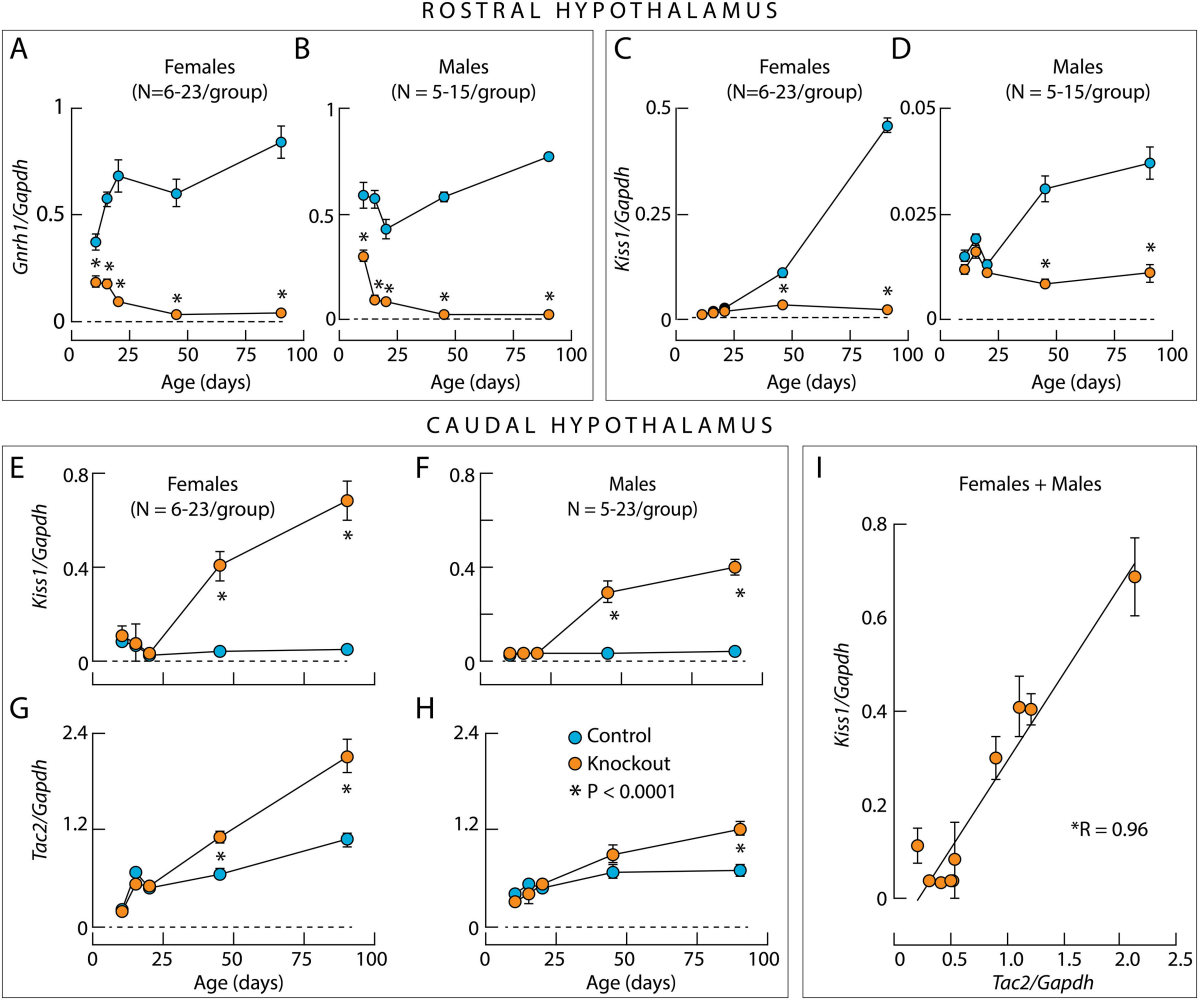
Developmental gene expression profile in the rostral and caudal hypothalamus of control and knockout mice. **(A–D)**, *Gnrh1* expression **(A, B)** and *Kiss1* expression **(C, D)** in the rostral hypothalamus of both sexes. Note the 10-fold difference in *Kiss1 expression of* control and knockout female **(C)** and male **(D)** mice. **(E–H)**, Facilitation of *Kiss1* expression **(E, F)** and *Tac2* expression **(G, H)** in female **(E, G)** and male **(F, H)** knockout mice in the caudal hypothalamus. **(I)**, Linear correlation between *Tac2* and *Kiss1* expression in the caudal hypothalamus of female and male knockout mice. Asterisks indicate significant (P < 0.0001) differences between pairs **(A–H)** using *post hoc* Šídák’s multiple comparisons test after one-way ANOVA, and for the correlation coefficient R using linear regression calculation. The legend in **(H)** applies to all panels. For rostral hypothalamus, female and male data include 6–23 and 5–15 animals for each point, respectively. For caudal hypothalamus, female and male data include 6–23 and 5–19 animals for each point, respectively. For clarity, individual data points for each mouse included in this analysis are not shown.

**Figures 7A, B** show the developmental profile of *Gnrh1* expression in the rostral hypothalamus. In the control group, *Gnrh1* expression was at comparable levels in females and males, but with somewhat different developmental profiles. In females, there was a prepubertal increase in expression of this gene, which was not evident in males. In the knockouts, there was a progressive loss of *Gnrh1* expression in both females and males, which was virtually undetectable in PND45 and PND90. In all ages, a difference between *Gnrh1* expression in the controls and knockout groups was highly significant (P< 0.0001). These results clearly indicate that the loss of GnRH immunoreactivity in adult knockouts reflects a decrease in *Gnrh1* expression in both sexes, further supporting the validity of the qRT-PCR assay for quantifying developmental changes in GnRH neuronal system.

Unlike *Gnrh1* expression, *Kiss1* expression in the rostral hypothalamus was sex-specific, with approximately 10-fold higher expression in females than in males. In both sexes, there was a significant increase in *Kiss1* expression with age (**Figures 7C, D**). *Kiss1* expression was similar in knockout mice and control mice up to PND20, but *Kiss1* expression was not increased in knockout females at PND45 and PND90. In the caudal hypothalamus of both sexes, *Kiss1* expression was similar in control and knockout mice up to PND20. However, in knockouts, *Kiss1* expression increased significantly above control levels at PND45 and PND90 (**Figures 7E, F**F).

Since ARN kisspeptin neurons co-express *Tac2* and *Pdyn* to drive their autonomous activity to generate GnRH pulses (28), the expression of these genes was also examined. In both sexes, *Tac2* expression was similar up to PND20 in knockout and control groups. At PND45, *Tac2* expression was increased in knockout females compared to control females, but not in knockout males (**Figures 7G, H**), whose *Kiss1/Tac2* neuronal development was delayed compared to females (29). Accordingly, at PND90, there was a significant increase in *Tac2* expression in males as well. Moreover, there was a highly significant correlation between *Tac2* and *Kiss1* expression (**Figure 7I**). In both sexes, *Pdyn* expression was similar between controls and knockouts at all ages studied, indicating that its expression was not affected by gonadal steroids (**Supplementary Figure 1A**). These experiments suggest that the expression pattern of *Kiss1* and *Tac2*, but not *Pdyn* expression, reflects the status of gonadal steroidogenesis. Notably, in both sexes, the expression of *Mkrn3* in caudal hypothalamic tissue decreased during postnatal maturation with similar kinetics between controls and knockout groups (**Supplementary Figure 1B**). This indicates that *Mkrn3* expression, which is crucial for regulating the onset of puberty (30), is independent of gonadal steroid hormones and *Gnrh1*, the latter being consistent with the literature (31).

### GnRH neurons have altered morphology in infantile knockout mice

Because GnRH immunoreactivity is associated with *Gnrh1* expression, the differences in shape of GnRH neurons in PND10 PI4KA knockout may reflect a reduction in GnRH immunoreactivity in processes below detection by immunostaining rather than actual changes in the morphology of these cells. To test this hypothesis, we visualized GnRH neurons using the cell type-specific expression of tdTomato, which is known to be ubiquitously expressed following Cre recombination of the Rosa locus and to fill the entire cell. Using this system, we were able to quantitatively track GnRH neurons (double labeled) and cells which have lost their GnRH antigen (single labeled) in knockout mice.

**Figure 8:**
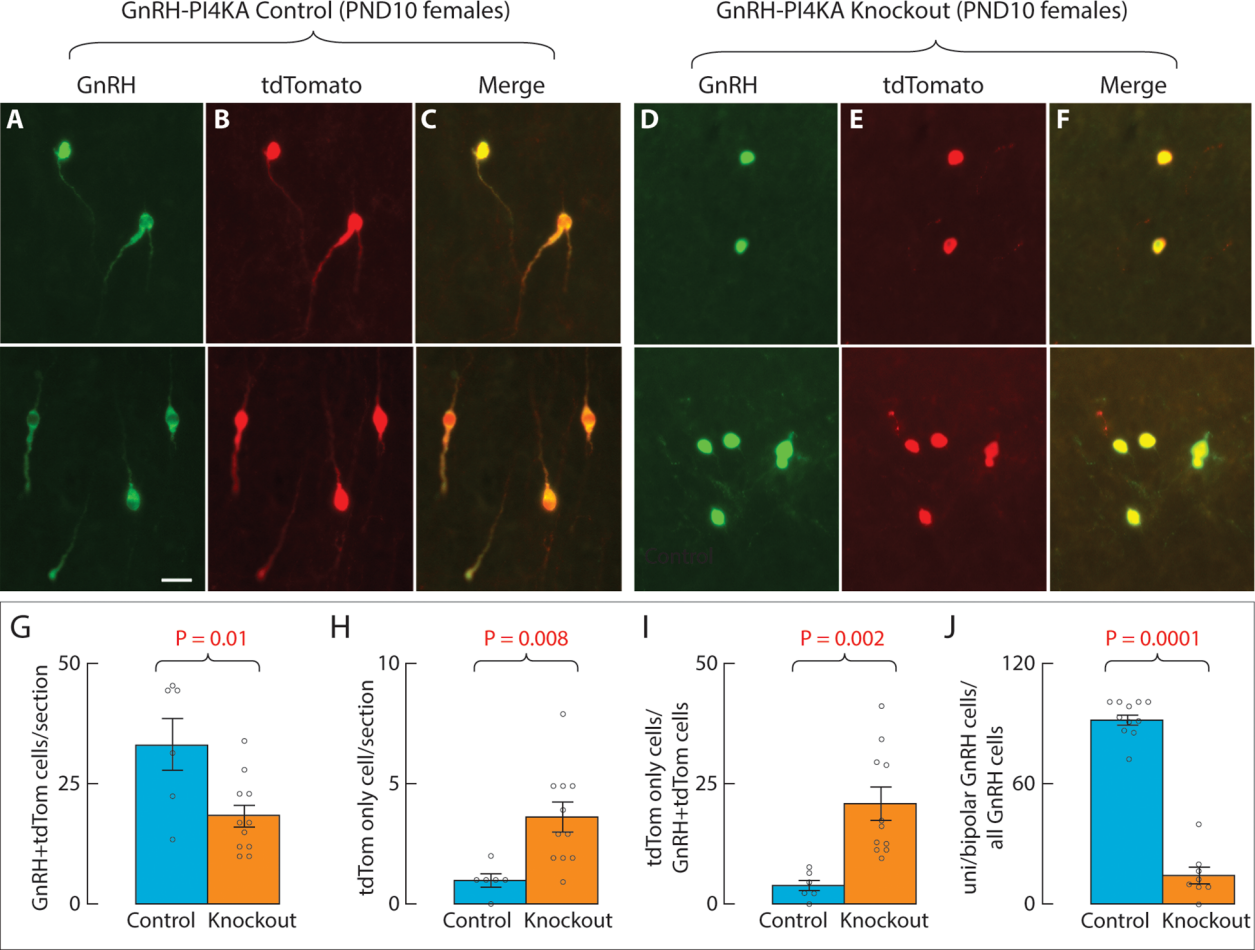
Dual identification of GnRH neurons in hypothalamic tissue from infantile control and knockout female mice. GnRH neurons were identified by GnRH immunofluorescence (green) and cell-type-specific tdTomato expression. For details, see Material and Methods. Representative images from the OVLT area are shown (upper and lower panels). **(A–C)**, Expression of GnRH **(A)**, tdTomato **(B)** and their merge **(C)** in control mice. **(D–F)**, Expression of GnRH **(D)**, tdTomato **(E)** and their merge **(F)** in knockout mice. Note the same shape of cells visualized by GnRH and tdTomato fluorescence in control and knockout mice. The horizontal bar at 20 μm applies to all panels. Three females were examined in each genotype. **(G–J)**, Cell type quantification. **(G)**, Number of GnRH-positive cells co-labeled with tdTomato. **(H)**, tdTomato-positive cells only. **(I)**, tdTomato-positive cells only expressed as a percentage of GnRH+tdTomato-positive cells. **(J)**, Number of unipolar/bipolar GnRH cells expressed as a percentage of GnRH-positive cells. Individual data points derived from multiple images per animal (N = 3 per genotype) are represented by small circles within and above the bars.

**Figure 9:**
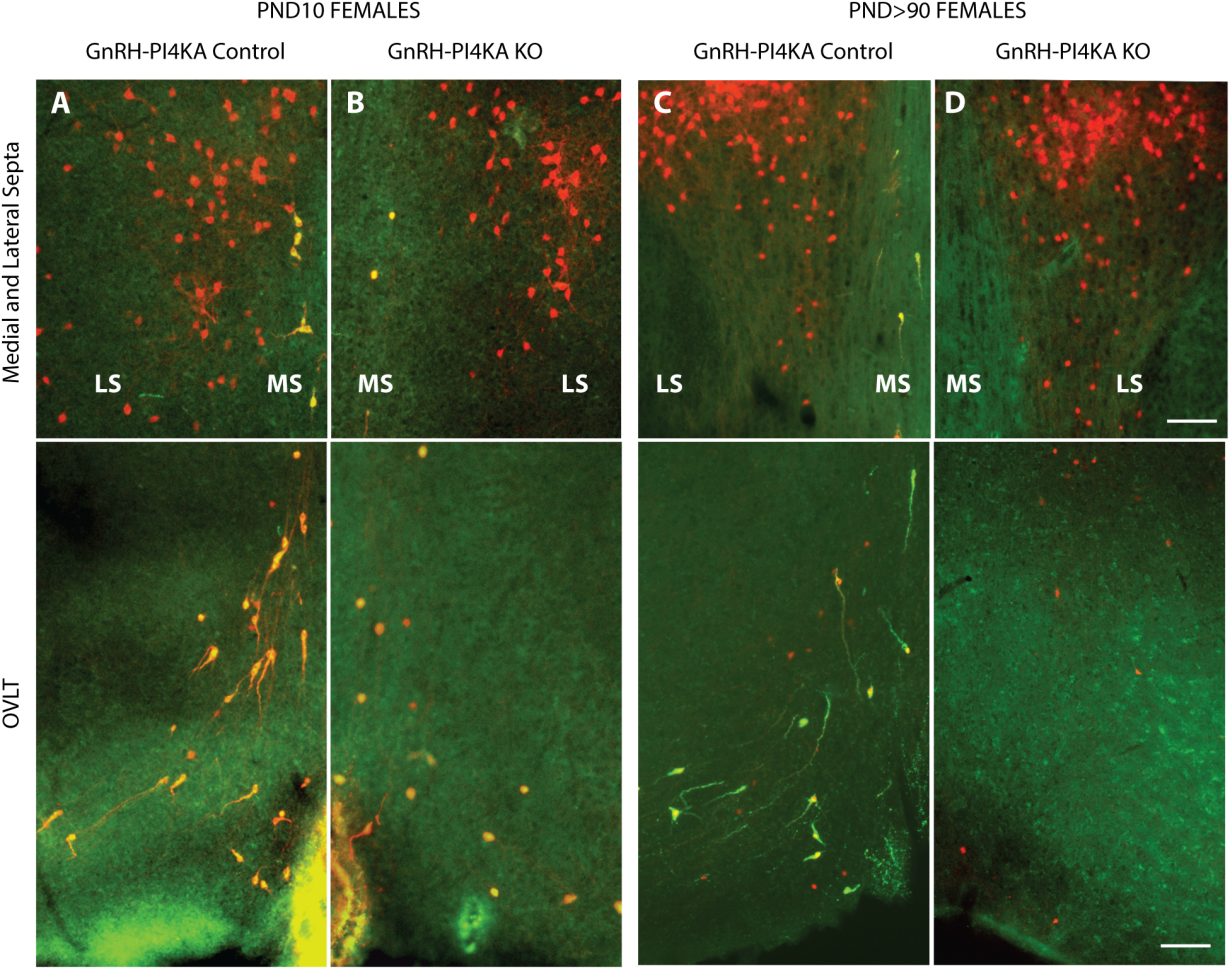
Comparison of tdTomato-expressing cells in the medial and lateral septa (upper panels) and in the OVLT region of the hypothalamus (lower panels) of infantile **(A, B)** (low magnification of **Figures 8A–F**) and adult **(C, D)** control and knockout female mice. Red indicates tdTomato-positive cells, and yellow indicates tdTomato+GnRH-positive cells. It should be noted that tdTomato-positive neurons are present in the lateral septum (LS) and are no affected by PI4KA knockout (**A–D**, upper panels). tdTomato+GnRH-positive cells present in the medial septum (MS) and OVLT region of the hypothalamus **(A, C)** show their bipolar morphology in controls, however these cells were round in infantile knockouts **(B)**, and disappeared in adult knockouts **(D)**. Horizontal bars at 100 µm apply to all panels Representative pictures are shown from three females examined for each genotype/age.

Experiments were performed on control (**Figures 8A–C**) and knockout (**Figures 8D–F**) females. Cell quantification (**Figures 8G–J**) was limited to the OVLT region, where ectopic tdTomato cells were less abundant (low magnification in **Figures 9A, B**, lower panels).

At PND10, sections from control females (N = 3) contained 33.2 ± 5.5 immunoreactive GnRH+tdTomato neurons, whereas sections from knockout females contained 18.3 ± 2.4 immunoreactive GnRH+tdTomato neurons (**Figure 8G**). Thus, only 55% of GnRH neurons were detected in knockout females, which is consistent with the 49% of *Gnrh1* expression in knockout females at PND10. Cells labeled with tdTomato alone were rare in control sections (1.0 ± 0.3/section), but were more frequently seen in knockout sections (3.6 ± 0.6/section) (**Figure 8H**). The same relationship was observed when tdTomato only cells were expressed relative to GnRH+tdTomato cells (**Figure 8I**).

The most obvious difference between GnRH+tdTomato cells from control and knockout sections was related their morphology, which was consistently observed in three independent preparations. In the control group, 92 ± 3% (N = 3 females) of cells were unipolar or bipolar with similar shapes when immunolabeled with GnRH antibody (**Figure 8A**), highlighted with tdTomato (**Figure 8B**), or when merged (**Figure 8C**). In the knockout group, both the GnRH antibody (**Figure 8D**), and tdTomato (**Figure 8E**) highlighted only 14 ± 4% (N = 3 females; **Figure 8J**) of cells as unipolar or bipolar, while the rest were round cells without any processes.

Therefore, based on the reduction in the number of GnRH+tdTomato cells, changes in their morphology, and the increase in the number of cells labeled with tdTomato alone, it is reasonable to conclude that PI4KA knockout in GnRH neurons impairs their survival.

### GnRH neurons, but not ectopic tdTomato cells, are lost in adult knockouts

During development, some cells, not derived from the olfactory placodes, transiently express *Gnrh1* in the lateral septum, and other areas (32). This transient expression is sufficient to induce *Gnrh1*-driven Cre expression, *Rosa* recombination, and tdTomato expression in cells other than neuroendocrine GnRH cells. Although *Gnrh1* expression is extinguished in these cells, *Rosa*-driven tdTomato expression persists through life, resulting in ectopic tdTomato-labeled cells, consistent with (33), who reported strong *Gnrh1*-Cre labeling within the lateral septum.

Taking advantage of this phenomenon, we were able to visualize ectopic tdTomato-labeled cells and tdTomato-labeled GnRH neurons, both PI4KA knockouts. Here, we compared tdTomato-expressing cells in the medial and lateral septum (**Figure 9**, top panels) and in the OVLT region of the hypothalamus (**Figure 9**, bottom panels) of infantile (*A* and *B*, N = 3 each) and adult (*C* and *D*, N = 2 each) control and knockout females, immunostained for GnRH. Red indicates tdTomato-positive cells only, and yellow indicates tdTomato+GnRH co-expressing cells. The lateral septum showed cells positive only for tdTomato, consistent with literature (32), and these cells were not affected by PI4KA deletion as they remained comparable to control females from PND10 to adulthood in knockout females (**Figure 9**, top panels). In contrast, the medial septum and OVLT region showed cells co-expressing tdTomato and GnRH with morphological changes in infantile knockouts, and these areas were devoid of tdTomato cells in adult knockouts (**Figure 9**, bottom panels). Together, this indicates that the morphological changes preceded the death of GnRH neuronsand that PI4KA knockout is not universally deleterious, but rather specific for neuroendocrine GnRH cells.

## Discussion

Here we show that knockout of PI4KA in GnRH neurons causes infertility in both females and males, reflecting the absence of puberty and underdeveloped gonads and reproductive organs. Since GnRH decapeptide controls the onset of puberty and fertility, these observations suggest a key role for PI4KA in the function of GnRH neurons. To further investigate this hypothesis, we performed immunolabeling studies, using adult controls and knockouts and a specific antibody against GnRH. These experiments revealed a lack of GnRH immunoreactivity of the knockouts. We also observed a significant reduction in *Gnrh1* expression, as assessed by qRT-PCR of hypothalamic tissue in adult females and males.

Without GnRH release, the expression of gonadotroph-specific genes in the pituitary gland should be reduced (34). This in turn should reduce the synthesis and release of gonadotropins, leading to underdeveloped gonads, absence of puberty, and infertility (35). Accordingly, we observed a significant reduction in *Lhb* and *Gnrhr* expression in adult pituitaries from both female and male adult knockouts. Expression of *Pomc*, *Tshb*, and *Gh1*, a marker gene for corticotrophs/melanotrophs, thyrotrophs, and somatotrophs, respectively, was not significantly affected, further indicating the specificity of the knockout. Within pituitary cells, *Spp1* is expressed only in gonadotrophs (24), and expression of this gene was significantly reduced in knockouts. The expression of this gene is not regulated by GnRH (36), but is stimulated by gonadal steroid hormones (37), which also stimulate *Prl* expression in lactotrophs (38). It is therefore reasonable to conclude that the reduced expression of *Spp1* and *Prl* reflects the lack of gonadal steroidogenesis in knockout females and males.

Previous studies have elucidated the embryonic steps involved in the development of GnRH neurons: their differentiation in the olfactory placodes, migration to the preoptic area and mediobasal hypothalamus, and projection to the median eminence (1). The establishment of connection between GnRH neurons and ARN kisspeptin neurons also occurs during the embryonic life, leading to the initiation of GnRH pulse generator activity in both females and males (39). The postnatal period is characterized by the connectivity of the GnRH system with RP3V kisspeptin neurons needed for puberty onset (40), and the preovulatory GnRH surge only in females (7).

Here we indirectly show that embryonic steps in the development of GnRH neurons are not critically affected by PI4KA knockout. In PND10 mice, we show that the distribution of GnRH neurons in the OVLT region was similar between controls and knockouts and that, albeit reduced, the median eminence was innervated by GnRH fibers. Our qRT-PCR experiments with PND3 females and males further revealed no difference in *Gnrh1* expression in knockout mice. However, *Gnrh1* expression was significantly affected by PND9 in knockout females and males. This reduction is consistent with the fact that *Gnrh1* expression and GnRH cell number are reduced and that most GnRH cell bodies lack processes. Although subtle effects of PI4KA knockout during embryonic life cannot be ruled out without a thorough examination of GnRH development, during the infantile period it becomes apparent that loss of PI4KA causes death of GnRH neurons.

Since GnRH secretion is regulated by kisspeptin (25, 41, 42), it was important to compare the status of two major hypothalamic populations of kisspeptin neurons in rodents, one in the RP3V, and the other in the ARN (43). The RP3V kisspeptinergic population is sexually dimorphic, being larger in females than in males. In contrast, the ARN kisspeptin neuron population does not exhibit sexual dimorphism (44). Here we show that RP3V kisspeptin immunoreactivity is higher in knockout females than in knockout males, but less abundant than in same-sex controls, consistent with reduced *Kiss1* expression. This finding suggests that the sex steroid status in PND15 knockout females, although likely impaired, is not as drastic as in gonadectomized PND15 females, in which RP3V immunoreactivity is completely eliminated (40).

Furthermore, we show that kisspeptin immunoreactivity in ARN is not sexually dimorphic in control and knockout groups, and that, in contrast to controls, knockouts display robust kisspeptin cell bodies, consistent with the lack of steroid negative feedback and the consequent increased expression of *Kiss1*. The loss of the dense fiber network accompanied by the appearance of kisspeptin cell bodies in the absence of negative feedback, previously described with this antibody (17, 21), is a known phenomenon that could be due to alterations in peptidergic transport (45), and therefore analysis of the accompanying expression is relevant. These data show that PI4KA knockout in GnRH neurons indirectly affects kisspeptin expression due to the loss of gonadal steroidogenesis. This is consistent with the fact that GnRH is not required for the establishment of kisspeptin populations (46).

To better understand the postnatal changes in GnRH neurons of knockouts, we further characterized the developmental expression profile of *Gnrh1* in the rostral hypothalamus and the expression profiles of *Kiss1* in both the rostral and caudal hypothalamus of control and knockout groups. The main finding of these experiments was the progressive decline in *Gnrh1* expression with postnatal age in both females and males, as well as the absence of the specific changes in *Kiss1* expression in the rostral and caudal hypothalamus in knockouts, changes that are normally associated with the establishment of steroid feedback as mice progress through puberty. Furthermore, we show parallelism in *Tac2* and *Kiss1* expression in knockouts in the caudal hypothalamus containing ARN kisspeptin neurons.

Gonadal steroid hormones regulate GnRH secretion and GnRH neurons lack steroid receptors, suggesting their indirect effects. Further studies revealed that RP3V kisspeptin neurons are responsible for the positive feedback of estradiol, while ARN kisspeptin neurons mediate the negative feedback of estradiol and testosterone (47–49). Consistent with this, our data show that sex steroid feedback is not established as knockouts grow. *Kiss1* expression remains low in the rostral hypothalamus due to the lack of steroid stimulation and increases in the caudal hypothalamus due to the lack of steroid inhibition. It is noteworthy that *Tac2* expression follows the same profile as *Kiss1* expression, as the *Tac2* gene is regulated in the same way (29).

These experiments could not clarify the nature of *Gnrh1* loss: irreversibly silenced *Gnrh1* expression or caused by premature death of GnRH neurons. To study the fate of GnRH neurons postnatally, it was necessary to identify them independently of their peptidergic phenotype. This was done using tdTomato reporter reflecting *Gnrh1*-driven Cre recombination, as described in Material and Methods section. Once recombination occurs in target cells, tdTomato is present until their death regardless of the nature of postnatal differentiation, as it is driven by the constitutively active ROSA26 promoter (50). Co-labeling of GnRH immunoreactive neurons with tdTomato confirmed the validity of our model for tracing GnRH neurons. The change in morphology of PND10 knockout GnRH neurons indicates the onset of degeneration of these cells. Furthermore, the loss of GnRH immunoreactivity in adult knockouts was accompanied by the loss of tdTomato labeling, indicating cell death.

In contrast, tdTomato cells in the lateral septum, which reflect transient expression of *Gnrh1* during development (32), i.e. ectopic tdTomato cells, persisted throughout life and were apparently unaffected by PI4KA knockout. As in GnRH neurons, PI4KA knockout in mouse gonadotrophs also caused infertility. However, in this case, infertility was not due to cell death, but to impaired GnRH receptor signaling, which requires PI(4,5)P2 production (17). Impaired GnRH receptor signaling led to dedifferentiation of existing gonadotrophs and reduced postnatal differentiation of new gonadotrophs from marginal zone stem cells (51). Therefore, cell death by PI4KA knockout is novel finding for neuroendocrine cells.

The relationship between phosphoinositides and cell death is a very complex issue and encompasses both the action of phosphoinositides as second messengers and their roles in the actin cytoskeleton and membrane dynamics, as well as their direct role in modulating cell death pathway (52). For example, phosphatidylinositol-3-kinase has been suggested to inhibit apoptosis (53), but to accelerate necrotic cell death (54). Lipotoxic disruption of NHE1 interaction with PI(4,5)P2 also accelerate apoptosis of proximal tubules (55), as does knockdown of PI4K2A or PI4KB (56). Dysregulation of PI4P production by knockout of PI4KA is also associated with increased lysosome-dependent cell death (57). Here, the loss of GnRH neurons in prepubertal animals due to PI4KA knockout is consistent with preliminary findings in other cell types, suggesting a direct or indirect cell type-specific role of PI4KA-derived phosphoinositides in the survival of these cells. Specifically, it has been shown that GnRH neuron survival depends on semaphorin 3E signaling associated with activation of the phosphatidylinositol-3-kinase pathway (58). However, further investigations are needed to identify the mechanism(s) by which the absence of PI4KA affects GnRH neuron survival, and which phosphoinositide, PI4, PI(4,5)P2, and/or PI(3,4,5)P3, accounts for these effects.

## Data Availability

The original contributions presented in the study are included in the article/**Supplementary Material**. Further inquiries can be directed to the corresponding author.

## Supplementary material

Supplementary Figure 1 Developmental gene expression profile in the caudal hypothalamus of control and knockout mice. **(A)**, *Pdyn* expression in females (left) and males (right). **(B)**, *Mkrn3* expression of both sexes. Means and SEM values are derived from 6–23 individual data points. There were no significant differences between control and knockout mice at any of the time points examined.
