# Supplementary figures and images for: PI4-kinase A is essential for survival of the GnRH neurons
Source: Front Endocrinol (Lausanne). 2026 May 5;17:1813498. doi: 10.3389/fendo.2026.1813498 (PMC13183541; doi:10.3389/fendo.2026.1813498)

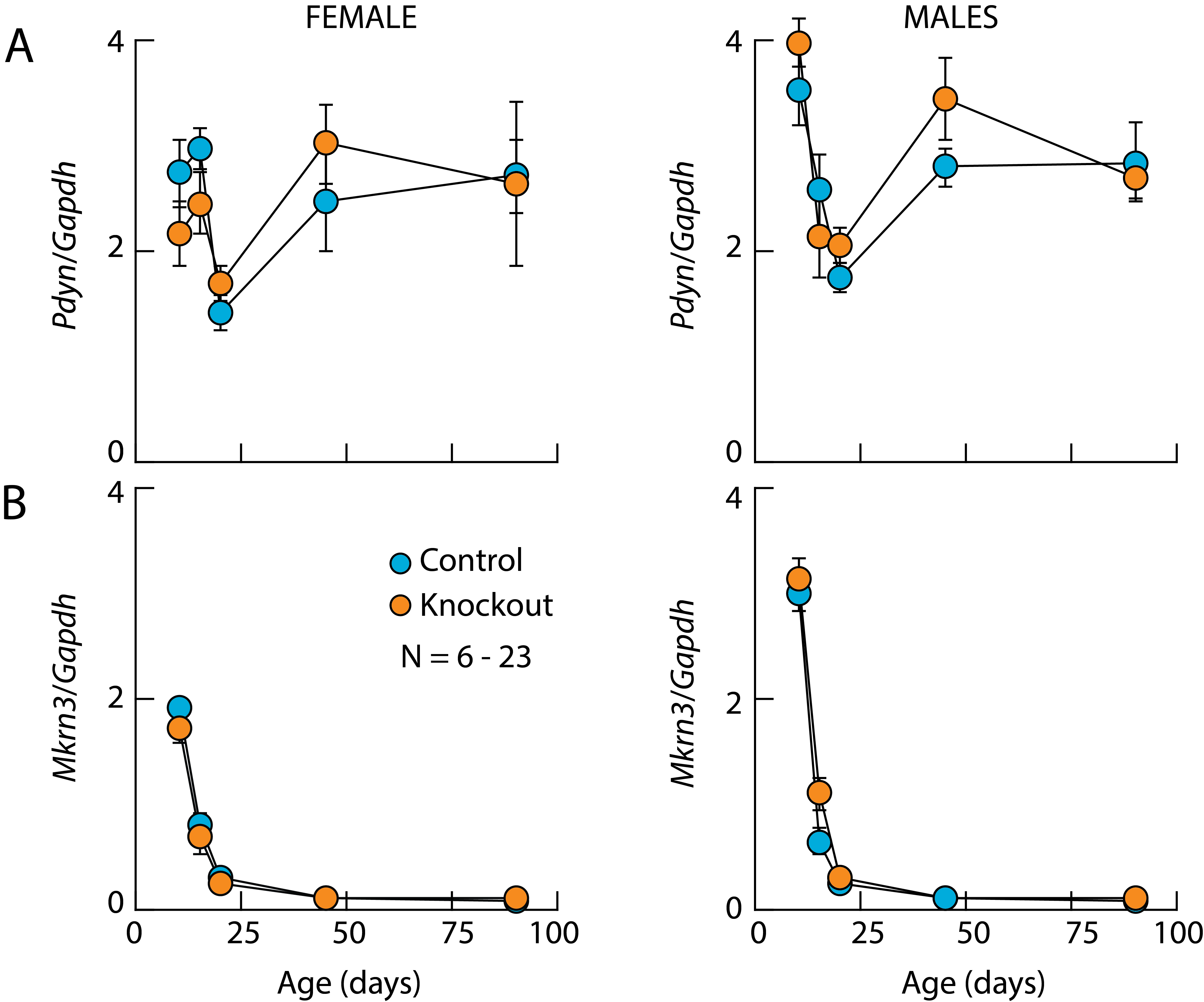

Supplement: Supplementary Figure 1 — Developmental gene expression profile in the caudal hypothalamus of control and knockout mice. (A), Pdyn expression in females (left) and males (right). (B), Mkrn3 expression of both sexes. Means and SEM values are derived from 6–23 individual data points. There were no significant differences between control and knockout mice at any of the time points examined. [file Image1.png]
